# Investigating the Citrus Aphid Species in Zhejiang, China: Morphometric Analysis and Genetic Distinctions

**DOI:** 10.1007/s13744-025-01299-x

**Published:** 2025-07-10

**Authors:** Jia Lü, You Li, Shunmin Liu, Muhammad Younas, Lianming Lu, Zhanxu Pu, Li Zhu, Guoqing Chen, Zhendong Huang

**Affiliations:** 1 The Citrus Research Institute of Zhejiang Province/Key Lab of Fruit and Vegetable Function and Health Research of Taizhou, Zhejiang Academy of Agricultural Sciences, Taizhou, Zhejiang China; 2 State Key Lab of Agricultural and Forestry Biosecurity, College of Plant Protection, Fujian Agriculture and Forestry Univ, Fuzhou, Fujian China

**Keywords:** Pests, Morphology, DNA, Genetic variation, Identification, Viruses

## Abstract

Aphids (Hemiptera: Aphididae) are well-known agricultural pests, affecting global crop production both through direct feeding and as the vector of multiple plant viruses. The classification of citrus aphid species was outdated and limited within the Zhejiang Province, China. A comprehensive investigation was conducted to determine the aphid species associated with citrus cultivars from 2019 to 2024. The present study included an analysis of occurrence rate, ecological contexts, and microscope slides photographs of apterous viviparous female, along with the key morphometric parameters such as body length, antenna, rostrum, hind tarsus, cauda, and siphunculi. Our morphological and molecular data revealed five species in this particular citrus region. Among the identified species, *Aphis* (*Aphis*) *gossypii* Glover and *A.* (*A.*) *spiraecola* Patch were the most prevalent, followed by *A.* (*A.*) *craccivora* Koch, *A.* (*Toxoptera*) *citricidus* (Kirkaldy), and *A.* (*T.*) *aurantii* Boyer de Fonscolombe. Furthermore, the genetic variation among aphid clades was assessed using Kimura 2-parameter (K2P) model. The results showed that citrus aphid species exhibits low intraspecific genetic variation and high interspecific genetic variation. Although the morphometric parameters of these aphid species are relatively similar, they can be distinguished through extensive morphological characteristics and cytochrome c oxidase subunit I (COI) fragment. Additionally, this study also addresses the historical and current distinctions among *A.* (*A.*) *spiraecola* and *A.* (*T.*) *citricidus*. These findings will significantly contribute to the ongoing research and management of citrus aphids in citrus plantations.

**Supplementary Information:**

The online version contains supplementary material available at 10.1007/s13744-025-01299-x.

## Introduction

The genus *Citrus* belonging to the family Rutaceae is one of the most widely cultivated fruit crop, with an annual production of 161.8 million tons worldwide (FAO 2023). It has been cultivated for more than 4000 years in the mainland of China for its nutritional and therapeutic values (Deng and Peng 2013). Zhejiang Province, located on the southeastern coast, is considered the top region for citrus cultivation, characterized by the longstanding history of agricultural development and germplasm resources. The citrus production has become important socio-economic activities and serves as one of the pillar industries in this region (Deng and Peng 2013; Xu 2019).

Citrus farming is usually threatened by hemipteran (aphids, scale insects, psyllids, etc.), and dipteran (fruit flies, etc.) insects. Out of these, aphids are deemed to be the most destructive sap-sucking pests in temperate regions. Multiple species of aphids including *A. spiraecola*, *A. gossypii*, and *A. aurantii* have been documented over the globe including Italy (Yahiaoui et al. 2009), Spain (Marroquín et al. 2004), Portugal (Paiva et al. 2024), and India (Ghosh et al. 2015). It has been documented that 25 species of aphids destroy citrus globally (Viggiani 1988; Jacas et al. 2010). Recently, Mathioudakis et al. (2025) also reported the existence of three aphids putative viruliferous species on citrus including *A. spiraecola*, *A. gossypii*, and *T. aurantii* from western Crete, Greece.

Citrus aphid species are seasonal pests, which are most prevalent in spring, early summer, and autumn. They usually form high-density colonies on citrus trees, cause direct damage to new shoots, contaminate the fruit, and result in early defoliation of leaves. Indirectly, they produce honeydew, ensuring the occurrence of sooty mold disease, and transmit citrus viruses (Deng and Peng 2013; Xu 2019). They have a major negative impact on citrus tree health, and reduce fruit production. Citrus aphid species have been studied in Zhejiang Province from 1993 to 1996 (Chen et al. 1993; Li et al. 1996; Ye et al. 1996a, b), and now the citrus management has created various uncertainties about the presence of current species due to changing climate, geographical ranges, diversity, composition, and structure of ecological communities (Altermatt 2010; Brose et al. 2012; Dong et al. 2013; IPCC 2014; Behi et al. 2019; Liu et al. 2021; Mathioudakis et al. 2025).

This paper examines the citrus aphid species in Zhejiang Province, China, with the aim of providing valuable insights about the citrus aphid species for disease management, epidemic prognosis, and the promotion of the future research on the relationship between aphids and citrus.

## Material and methods

### Sampling sites and collection

**Fig. 1:**
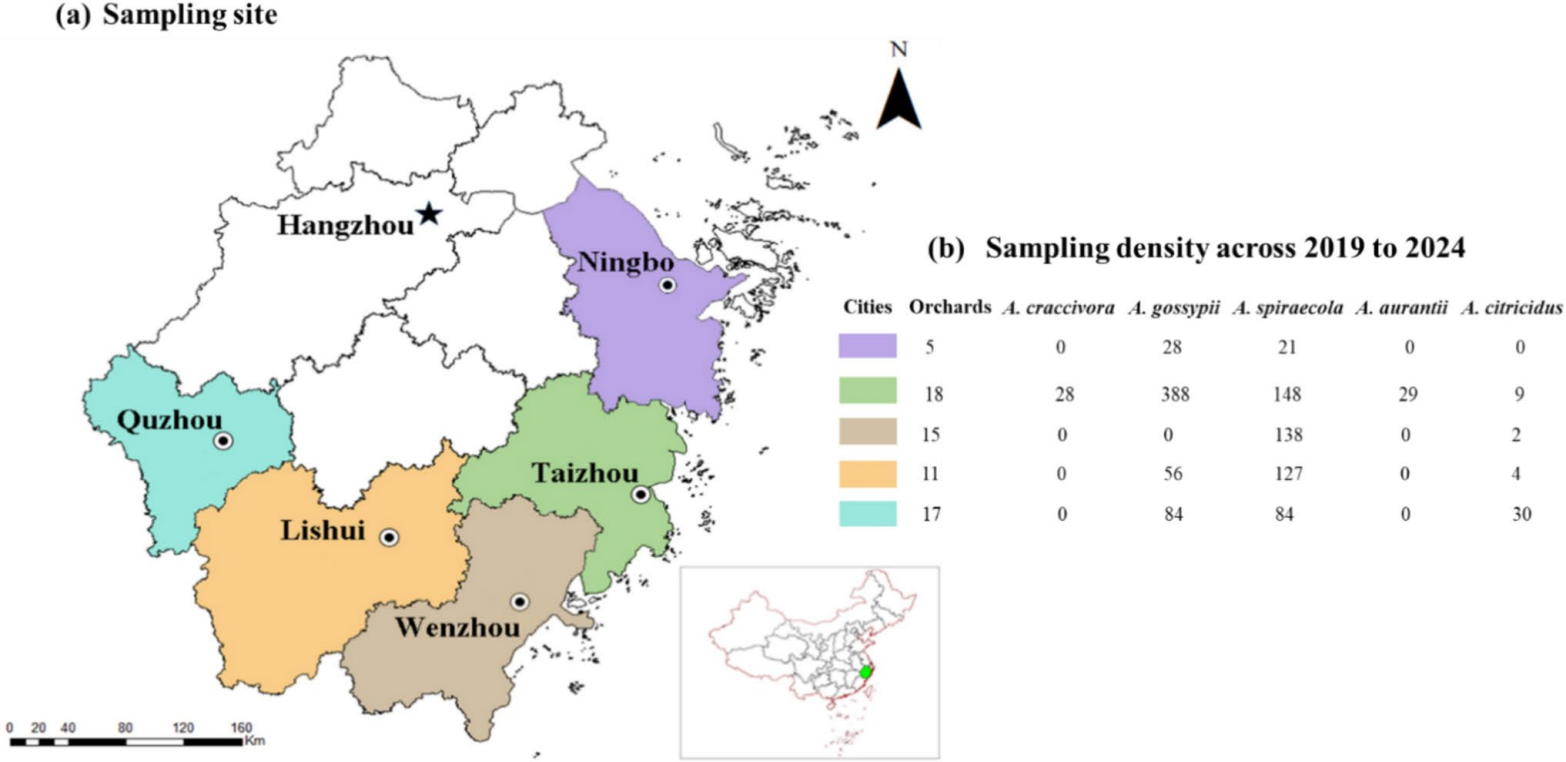
**a** Distribution of sampled citrus orchards across five citrus-planting cities in Zhejiang, China, and (**b**) the sampling density of the citrus aphid species across studied area during 2019 to 2024

A total of approximately 1200 aphid samples, with each sample representing an aphid population from a single flush, were collected across five major citrus-growing cities (Lishui, Ningbo, Quzhou, Taizhou, Wenzhou; Fig. 1) in Zhejiang Province during the citrus-growing seasons from 2019 to 2024. All samples were preserved in ≥ 99.7% ethanol and kept in − 20 °C to ensure the integrity for further morphological measurement and molecular analysis.

### Slide samples preparation and preservation

Every aphid species with apterous viviparae was selected to make microscope slide specimens [*Aphis* (*Aphis*) *craccivora*, *n* = 12, *Aphis* (*Aphis*) *gossypii*, *n* = 15, *Aphis* (*Aphis*) *spiraecola*, *n* = 14, *Aphis* (*Toxoptera*) *aurantii*, *n* = 11, *Aphis* (*Toxoptera*) *citricidus*, *n* = 12], following the descriptions of Li (1990) and Xu et al. (2017). The preserved samples and the permanent slides of the citrus aphid species were kept in the Plant Protection Research Office, The Citrus Research Institute of Zhejiang Province, Zhejiang Academy of Agricultural Sciences.

Morphological terminology and species nomenclature used in this study follow the widely accepted taxonomic classification by Zhang and Zhong (1983), Blackman and Eastop (2006), and Lagos et al. (2014). However, morphometric data and photographic records were generated from freshly collected specimens examined during the present survey. Observations regarding coloration and seasonal morphological variation such as grass green forms typically observed in spring and autumn, and pale whitish-yellow forms in the summer are based on original field observations conducted in contemporary study. A total of the twelve morphological parameters were analyzed for each citrus aphid species, including antennal segments III, IV, V (ANT III, ANT IV, ANT V); length of the *processus terminalis* of the last antennal segment (PT); basal length of last antennal segment (BASE); body length (BL); caudal length; basal width of the cauda; length of the siphunculi (SIPH); basal width of the siphunculi; the combined length of segment IV and V of rostrum (R IV + V); and the length of the second segment of the hind tarsus (HT II). The detailed morphometric data for five citrus aphid species can be found in the supplementary materials (Supplementary Table S1 to Table S5).

Photographs and data processing were executed using the Microsoft 365 for the occurrence rate pie chart, whereas the ecological photos of the citrus aphid species were captured with a digital camera (Canon, MF4410) equipped with a 600 × 600 dpi optical resolution and a 24-bit color depth, ensuring clear and detailed imagery. The slide samples images, and the morphometric parameters were captured by the Nikon stereo microscope (Nikon, SMZ25) which features a high-precision zoom range and an integrated digital imaging system for accurate capture of diagnostic traits. The resulting images were post-processed and annotated using Adobe Photoshop CC 2019 to enhance clarity and standardize scale bars, ensuring consistency in visual presentation across all morphological datasets.

### DNA extraction and polymerase chain reaction (PCR)

DNA was extracted from a citrus aphid population with a single aphid using Ezup Column Animal Genomic DNA Purification Kit (Sangon Biotech, China) following the manufacturer’s protocols; the part samples and the detail information from our collections that have been chosen for DNA extraction are listed in Supplementary Table S6. The PCR setup consisted of a total volume of 30 µl containing 15 µl of the master mix, 10.5 µl of dd H_2_O, 1.5 µl of each primer [LCO 1490: 5′-GGTCAACAAATCATAAAGATATTGG-3′ and HCO 2198: 5′—TAAACTTCAGGGTGACCAAAAAATCA-3′] at 5 µmol/l, and 1.5 µl of DNA. The cycling conditions included initial denaturation at 94 °C for 4 min, followed by 40 cycles of denaturation at 94 °C for 30 s, annealing at 50 °C for 50 s, extension at 72 °C for 1 min, and a final elongation cycle of 5 min at 72 °C (Hebert et al. 2003). PCR products were sequenced by Sangon Biotech (China).

### Molecular analysis

The initial sequences were analyzed using software DNASTAR (Burland 1999) and verified with the BLASTN algorithm from National Center for Biotechnology Information (NCBI). Additional related sequences were retrieved from GenBank (Supplementary Table S7). All the aligned sequences were trimmed to the 661 bp for mitochondrial cytochrome c oxidase subunit I (COI) gene fragment, and saturation analysis was performed to assess evolutionary accuracy. The aphid phylogenetic tree based on COI sequences was reconstructed using Neighbour-Joining (NJ) analysis in MEGA 7, with *Myzus persicae* (KY509874) and *Diuraphis noxia* (AF548467) as outgroups. The clade supports were performed with the standard nonparametric bootstrap analysis of 1000 replicates, and the genetic distances calculated under Kimura 2-parameter (K2P) model (Kumar et al. 2016).

## Results

### Prevalence of citrus aphid species in Zhejiang Province

**Fig. 2:**
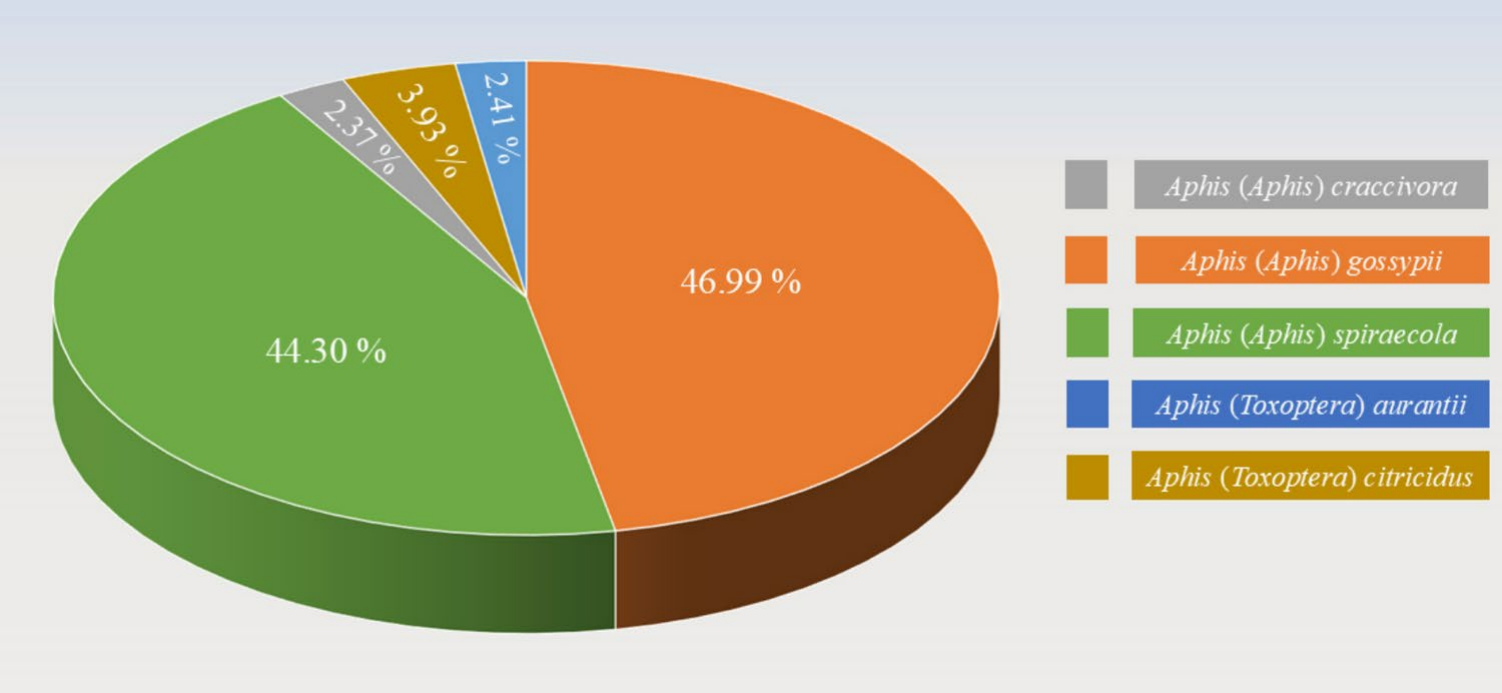
The occurrence rate of the citrus aphid species in Zhejiang Province, China

The identification of citrus aphid species and their morphometric characteristics was conducted based on the samples collected from the citrus orchards in Zhejiang Province. A total of five citrus aphid species, all belonging to two subgenera (*Aphis* and *Toxoptera*) within the genus *Aphis*, were identified, including *A.* (*Aphis*) *craccivora* Koch (2.37%), *A.* (*A.*) *gossypii* Glover (46.99%), *A.* (*A.*) *spiraecola* Patch (44.30%), *A.* (*Toxoptera*) *aurantii* (Boyer de Fonscolombe) (2.41%), and *A. (T.) citricidus* (Kirkaldy) (3.93%) (Fig. 2). However, *A.* (*A.*) *gossypii* and *A.* (*A.*) *spiraecola* were the most dominant species in this region. The identification and characteristics of citrus aphid species were based on the references provided by Zhang and Zhong (1983), Blackman and Eastop (2006), and Yu and Wang (2019).

### Morphometric identification of citrus aphid species

Citrus aphid species and their characteristics were referenced from Zhang and Zhong (1983), Blackman and Eastop (2006) and Yu and Wang (2019):

*Aphis* (*Aphis*) *craccivora* Koch, 1854

Common name: cowpea aphid, which colonize various plants, particularly leguminous crops.

**Fig. 3:**
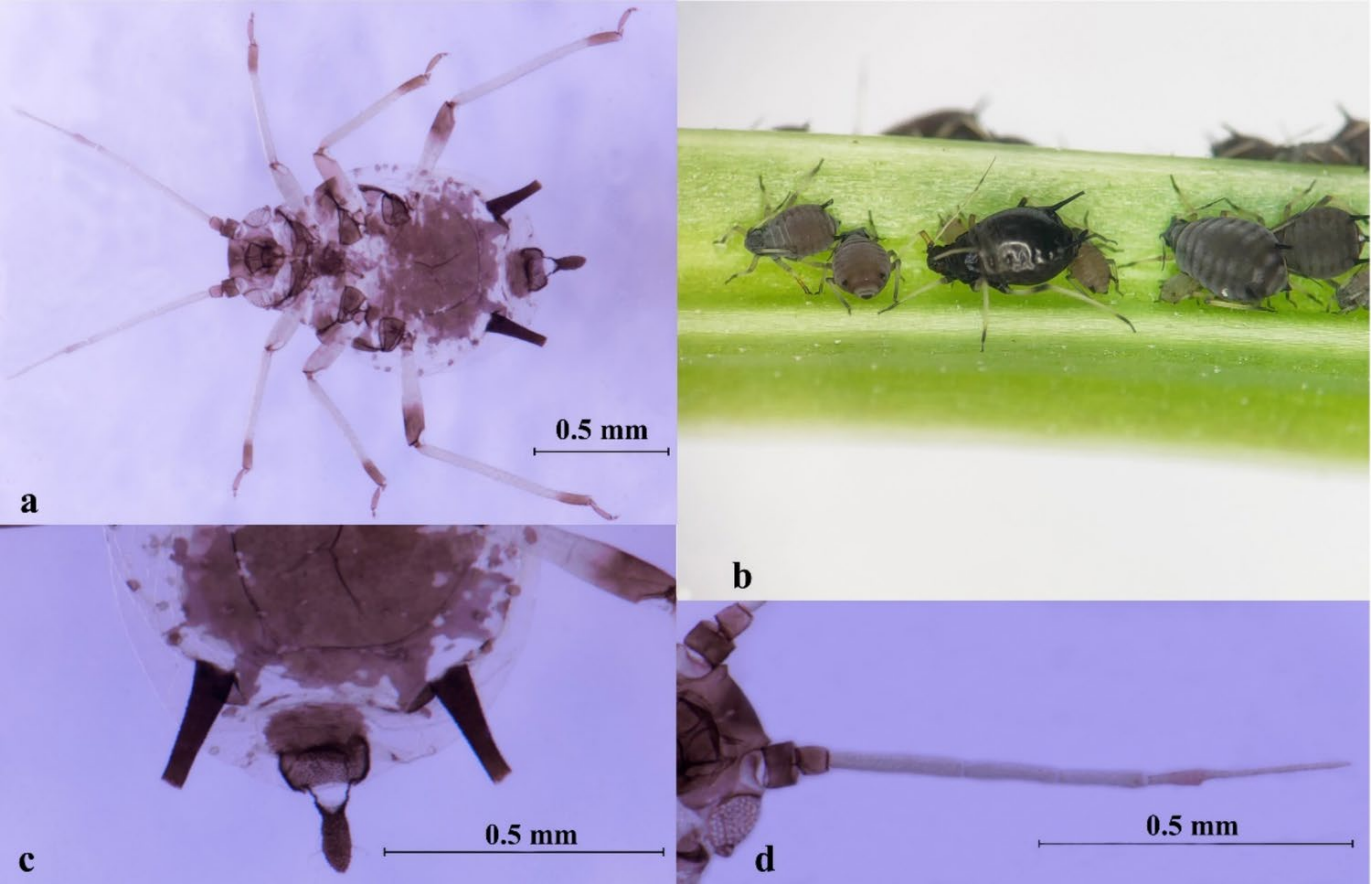
*Aphis* (*Aphis*) *craccivora*. **a** Photo of microscope slide; **b** colony on citrus; **c** antenna; **d** siphunculi and cauda

Description: BL about 1.35 ~ 1.60 mm (*n* = 12). Apterous viviparous morphs black, paint black or thick purple, shiny. Antennae segment 1 and 2, and the end dark brown, pale in the remaining segments. The end femur, tibia, and tarsus dark brown. Antennae (ANT) tubercles weakly developed, ANT PT/BASE about 1.91 ~ 3.11. R IV + V mostly 0.82 ~ 1.33 × HT II. Dorsal abdomen with an extensive black shield and a solid black patch. SIPH black, more than 3.00 × their basal width, and 1.60 ~ 2.05 × cauda. Cauda black, as black as SIPH, with distal part tapering, more than 1.50 × longer than its basal width (Fig. 3, Table S1).

*Aphis* (*Aphis*) *gossypii* Glover, 1877

Common name: cotton aphid, melon aphid, cotton-melon aphid. This polyphagous pest exhibits wide host range.

**Fig. 4:**
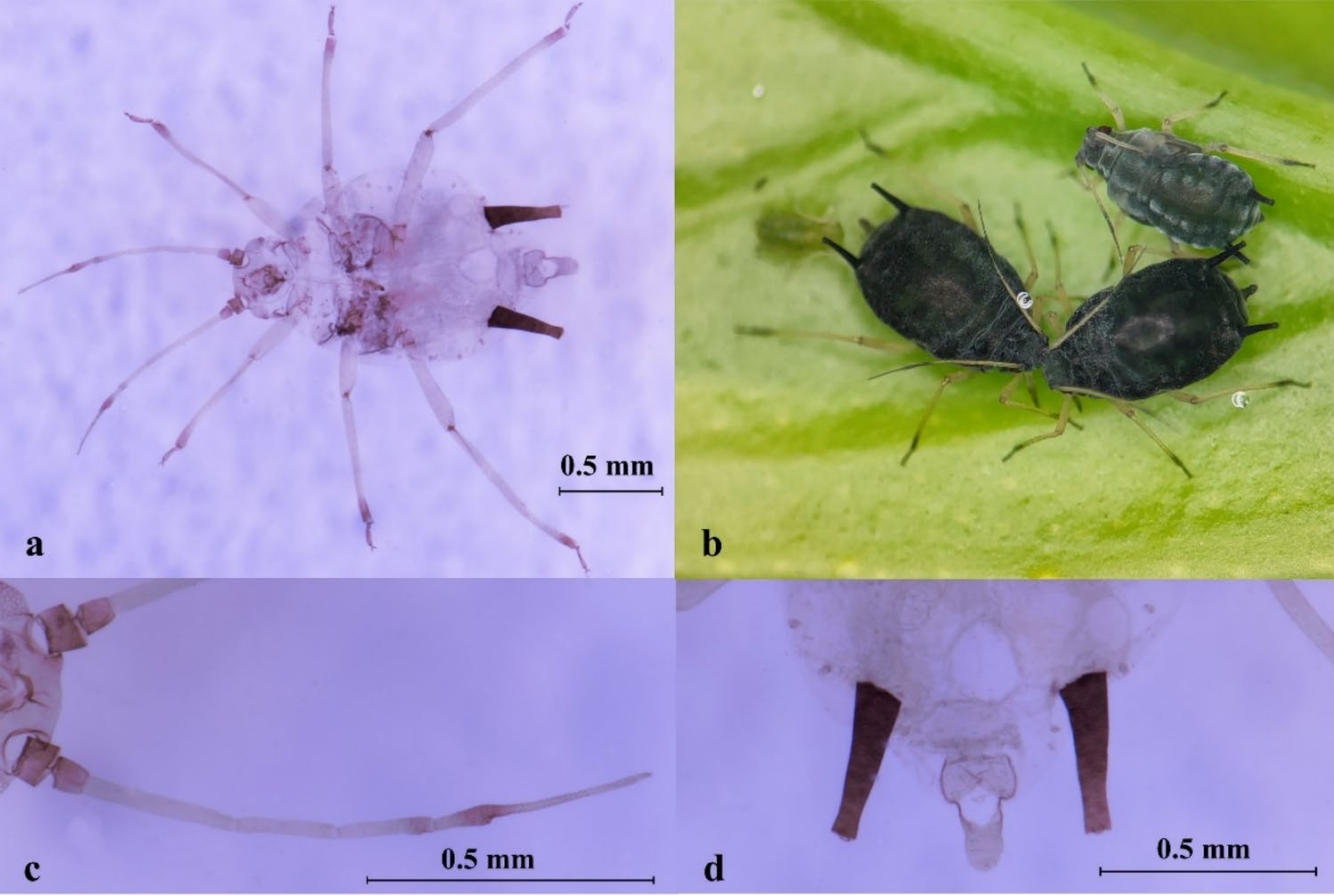
*Aphis* (*Aphis*) *gossypii*. **a** Photo of microscope slide; **b** colony on citrus; **c** antenna; **d** siphunculi and cauda

Description: BL about 1.31 ~ 1.49 mm (*n* = 15). Apterous viviparous morphs with varying body colors, commonly dark blackish green, grass green (larger species usually in spring and autumn), or pale whitish yellow (small species usually in hot summer). Antennae segment 1, 2, 6 and the distal 2/5 of segment 5 black. The end 1/5 of tibia and tarsus dark brown. ANT tubercles weakly developed, not projecting beyond middle part of frons in dorsal view, usually 6-segmented, sometimes 5-segmented in small individuals, and ANT PT/BASE about 2.45 ~ 3.29. R IV + V 0.09 ~ 0.11 mm, and less than 1.50 × HT II. Dorsal abdomen unpigmented, without any clearly defined dark markings. SIPH uniformly dark, which has no constriction, and clearly darker than cauda, more than 0.10 × BL, and tapering from base to flange, with no trace of swelling. Cauda pale to dusky, without any constriction (Fig. 4, Table S2).

*Aphis* (*Aphis*) *spiraecola* Patch, 1914

Common name: green citrus aphid, *spiraea aphid*. A cosmopolitan pest with wide range of hosts, and recognized as the major pest of citrus crops.

**Fig. 5:**
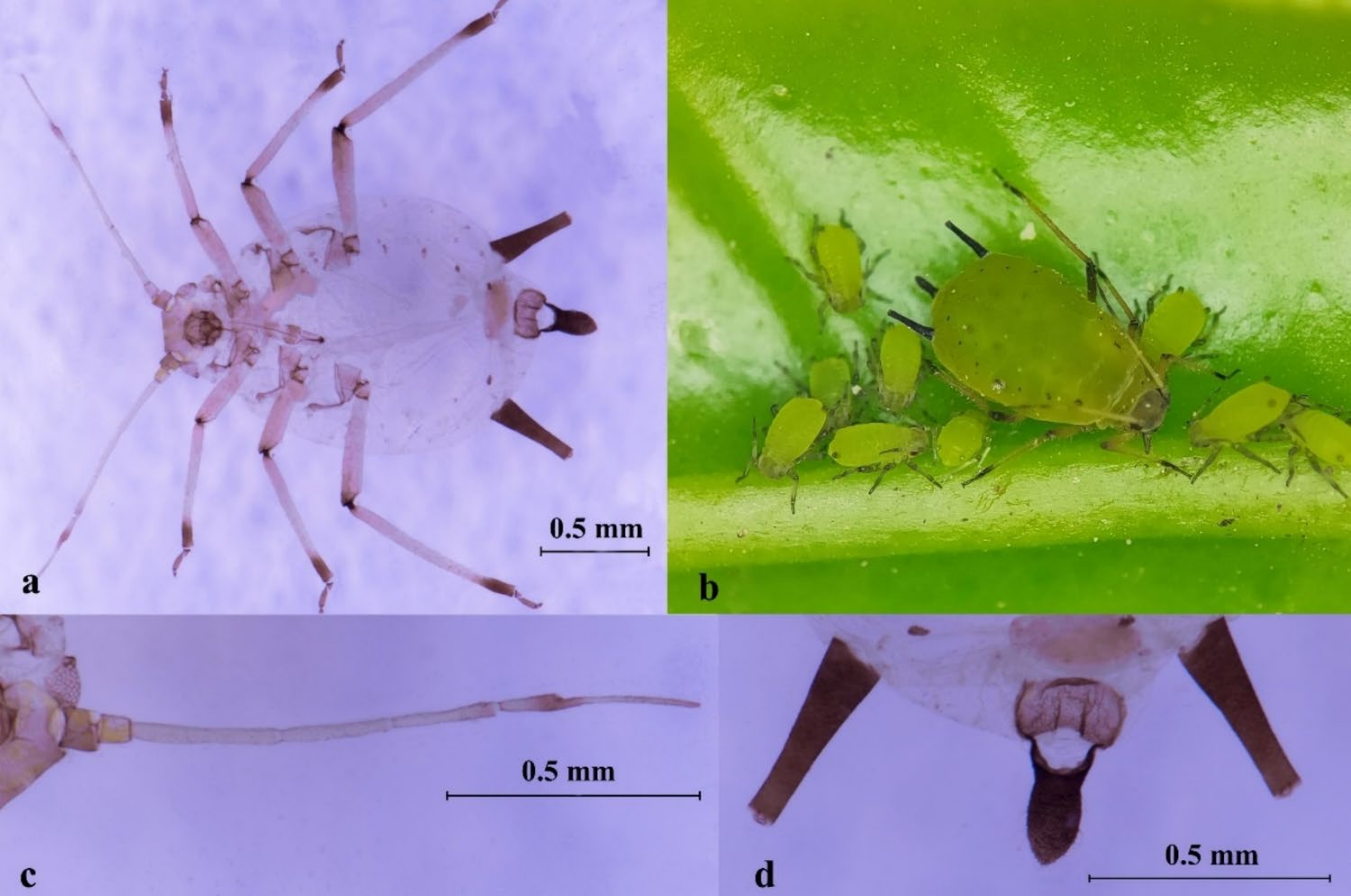
*Aphis* (*Aphis*) *spiraecola*. **a** Photo of microscope slide; **b** colony on citrus; **c** antenna; **d** siphunculi and cauda

Description: BL about 1.41 ~ 1.62 mm (*n* = 14). Apterous viviparous morphs bright greenish yellow to apple green. Antennae segment 1 and 2, the 1/3 basal part of segment 6, and the end of the femur, the tibia, and the tarsus dark brown, pale with the rest. ANT tubercles weakly developed. ANT PT/BASE less than 3.0, and ANT III 0.70 ~ 0.93 × SIPH. R IV + V 1.00 ~ 1.22 × HT II. Dorsal abdomen without any dark markings. SIPH dark. Cauda black like SIPH, usually has an evident constriction between basal and distal part (Fig. 5, Table S3).

Remarks: multiple studies have historical ambiguity about this aphid species as “*Aphis citricola*” van der Goot, 1912 (the details are in the discussion part).

*Aphis* (*Toxoptera*) *aurantii* (Boyer de Fonscolombe, 1841)

Common name: black citrus aphid. Polyphagous.

**Fig. 6:**
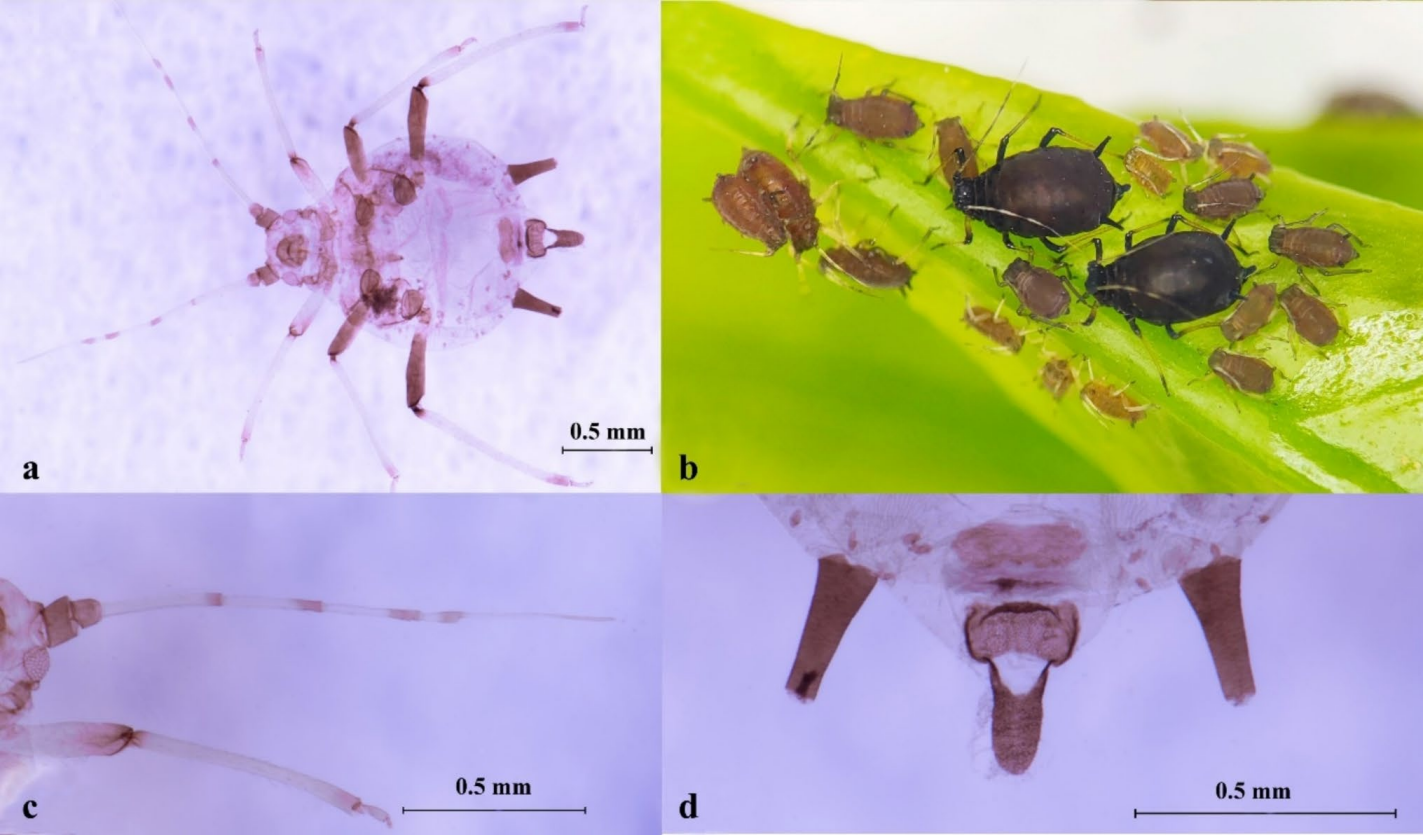
*Aphis* (*Toxoptera*) *aurantii*. **a** Photo of microscope slide, ventral view; **b** colony on citrus; **c** antenna; **d** siphunculi and cauda

Description: BL about 1.42 ~ 1.69 mm (*n* = 11). Apterous viviparous morphs black to dark brown. Antennae segment 1, 2, the end of segment 3, 4, 5, and the 1/2 basal part of segment 6 black, pale with the rest. The femur of middle-leg and hindleg, the base and the distal part of the tibia black, pale with the rest. ANT tubercles weakly developed, PT/BASE 4.10 ~ 4.89. R IV + V 1.33 ~ 1.86 × HT II. SIPH and cauda entirely dark. SIPH 1.00 ~ 1.53 × cauda (Fig. 6, Table S4).

*Aphis* (*Toxoptera*) *citricidus* (Kirkaldy, 1907)

Common name: brown citrus aphid. An oligophagous aphid mainly on Rutaceae plants, and which is a principal virus vector of citrus.

**Fig. 7:**
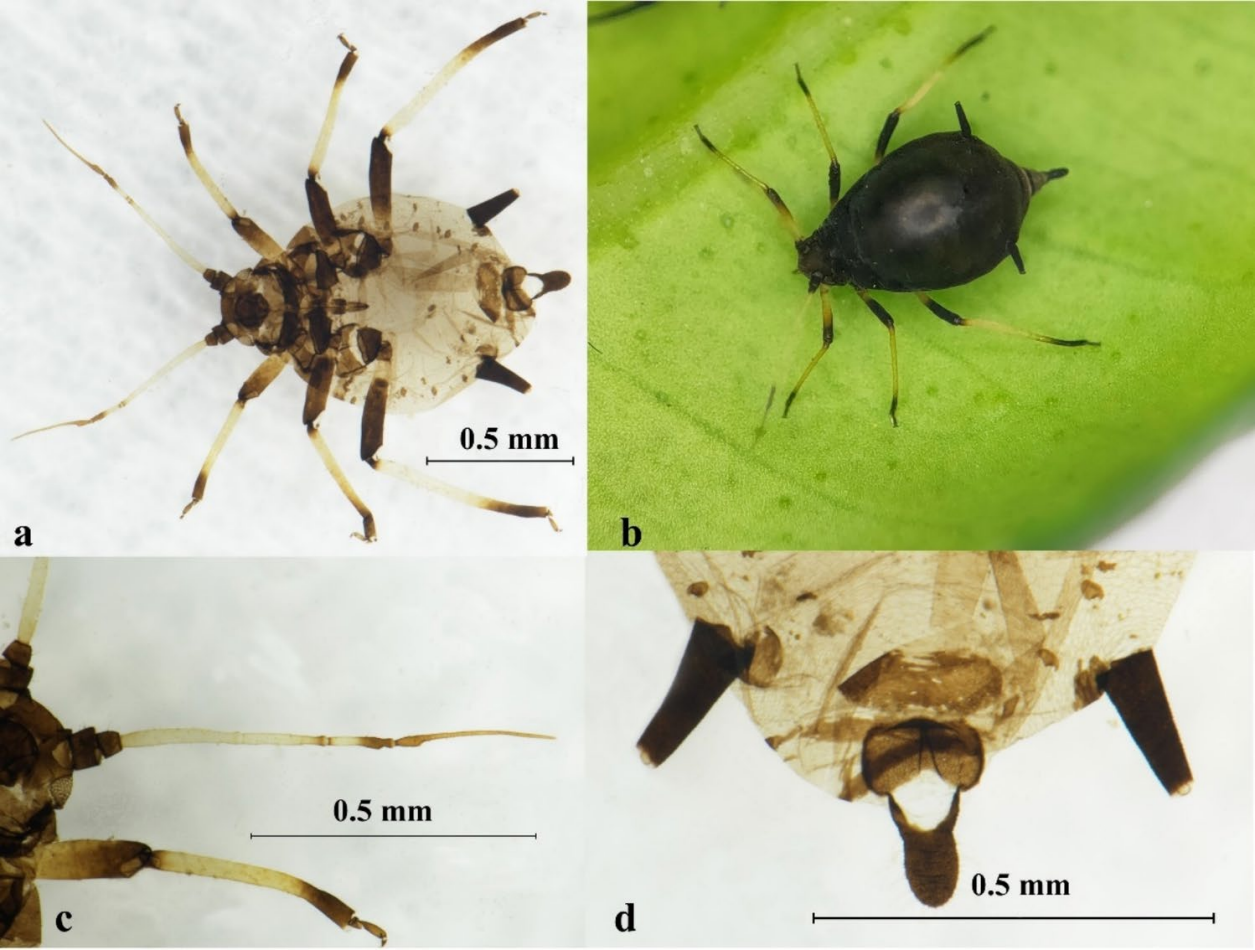
*Aphis* (*Toxoptera*) *citricidus.* **a** Photo of microscope slide, ventral view; **b** adult; **c** antenna; **d** siphunculi and cauda

Description: BL about 1.96 ~ 2.24 mm (*n* = 12), and usually more than 2.0 mm. Apterous viviparous morphs black to dark brown, shiny. Antennae segment 1, 2, the end of segment 4, the end 1/2 of segment 5, and segment 6 black, pale with the rest. The 1/2 basal part of profemur, and the 2/3 middle part of each tibia pale, black with the rest. ANT tubercles weakly developed, PT/BASE 4.55 ~ 6.22. Thoracic segments often partly sclerotised. R IV + V 1.17 ~ 1.60 × HT II. SIPH and cauda both dark, SIPH 1.10 ~ 1.65 × cauda (Fig. 7, Table S5).

Remarks: the aphid species *Aphis* (*Toxoptera*) *citricida* is an unjustified emendation of Dr. Stoetzel (Nafría et al. 2005).

### Phylogenetic analysis of mitochondrial gene (COI)

The COI fragments of 21 citrus aphid individuals were sequenced yielding a range of 671 to 704 bp. Following the trimming for alignment, 661 bp were used for the phylogenetic and cluster analysis. The results of transitions and transversions showed that the sequences did not reach saturation.

**Fig. 8:**
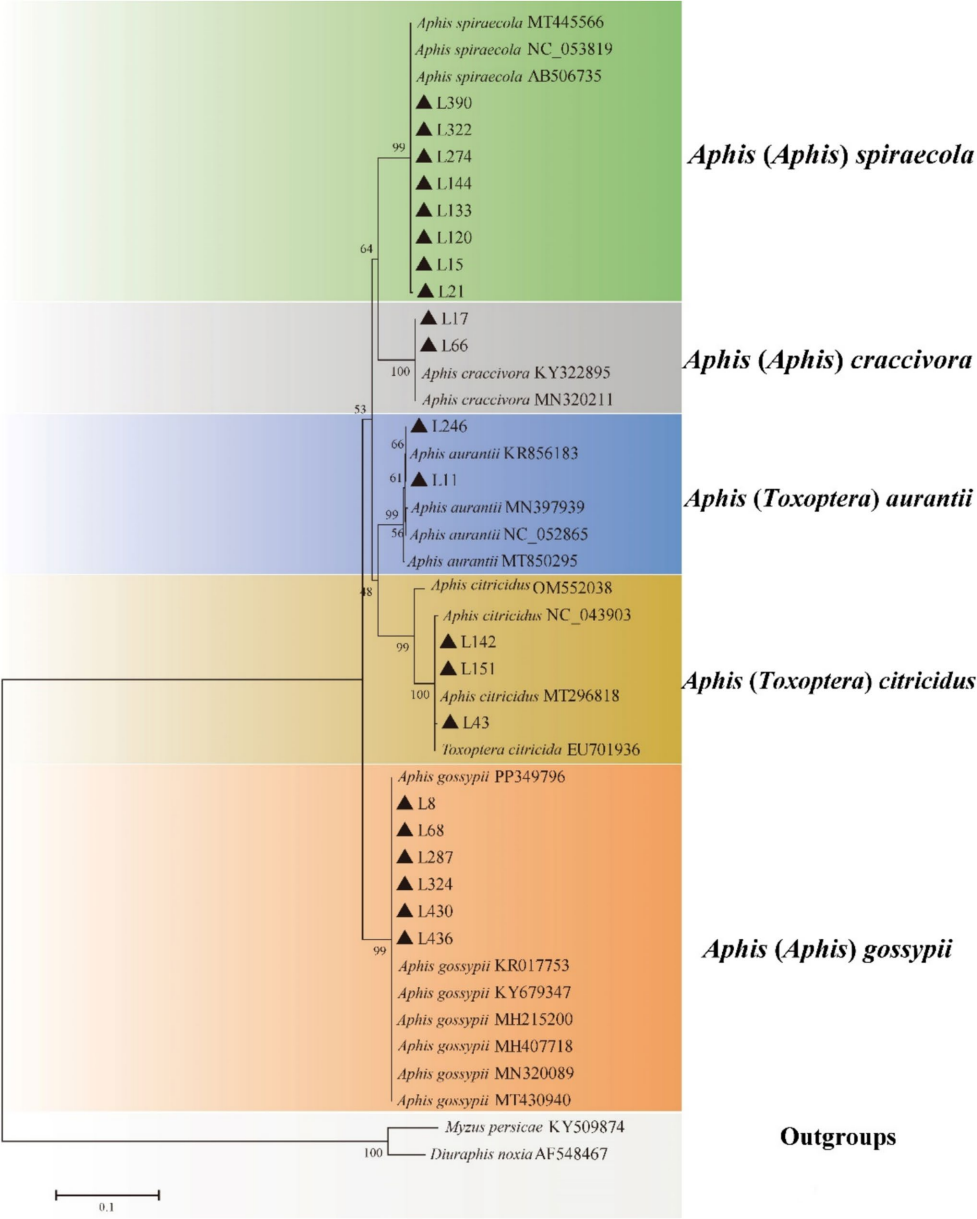
The Neighbour-Joining phylogenetic tree of citrus aphid samples based on the COI sequences; black triangles represent the samples collected in this study (Table S6)

The Neighbour-Joining analysis (Fig. 8) distinctly revealed five independent clades with strong support of *Aphis* (*Aphis*) *craccivora* (100, gray clade), *A.* (*A.*) *gossypii* (99, reddish orange clade), *A.* (*A.*) *spiraecola* (99, green clade), *Aphis* (*Toxoptera*) *aurantii* (99, blue clade), and *A.* (*T.*) *citricidus* (100, claybank clade). These molecular analyses support our morphological results, about the occurrence of five citrus aphid species in Zhejiang province.

The intraspecific genetic distances of the *Aphis* (*Aphis*) *craccivora* and = *A.* (*A.*) *gossypii* clade were 0.000, the *A.* (*A.*) *spiraecola* clade ranged from 0.000 to 0.002, the *Aphis* (*Toxoptera*) *aurantii* clade ranged from 0.000 to 0.003, and the *A.* (*T.*) *citricidus* clade ranged from 0.000 to 0.033. The interspecific distance of the *Aphis* (*Aphis*) *craccivora* clade was 0.819 to 0.821, the *A.* (*A.*) *gossypii* clade ranged from 0.783 to 0.809, the *A.* (*A.*) *spiraecola* clade ranged from 0.813 to 0.833, the *Aphis* (*Toxoptera*) *aurantii* clade ranged from 0.805 to 0.816, and the *A.* (*T.*) *citricidus* clade ranged from 0.803 to 0.845, compared with *Myzus persicae* and *Diuraphis noxia*, respectively*.* The genetic divergences within and between each clade of the aphids are provided in Supplementary Table S8 to S12, respectively.

## Discussion

Aphids are seasonal pests in citrus plantations, recognized for their detrimental effects in sap extraction, deforming leaves, and transmitting plant viruses (Hall and Albrigo 2007; Goggin 2007; Deng and Peng 2013; Bouvet et al. 2019). Present investigations found the occurrence of five citrus aphid species within two genera belonging to genus *Aphis* and two subgenus *Aphis* (*Aphis*), *Aphis* (*Toxoptera*). The classification of the citrus aphids was successfully substantiated by Neighbour-Joining (NJ) phylogenetic analysis, that revealed the bootstrap values ranges from 99 to 100, showing high confidence in the observed divergence among intra-species.

The genetic distances were assessed using Kimura 2-parameter (K2P) model, that demonstrated that *Aphis* (*Aphis*) *craccivora*, *A.* (*A.*) *gossypii*, and *Aphis* (*Toxoptera*) *aurantii* exhibited no intraspecific genetic variation within this geographical area. Additionally, *Aphis* (*Aphis*) *spiraecola* and *Aphis* (*Toxoptera*) *citricidus* showed minimum genetic variation of 0.002. However, the citrus aphid species in this area exhibits low level of intraspecific genetic variation.

The results of the contemporary study are in line with the findings of previous studies demonstrating multiple aphid species, including *Aphis gossypii*, *Aphis spiraecola*, *Toxoptera citricidus*, *Toxoptera aurantii*, *Myzus persicae*, *Sinomegoura citricola*, and *Toxoptera odinae* in the studied area. However, *Aphis craccivora* and *Lipaphis erysimi *have not been detected at this time (Chen et al. 1993; Li et al. 1996). Results are also supported by the findings of Behi et al. (2019) that *Aphis spiraecola* and *Toxoptera aurantii* were the abundant species in the early years in Tunisian fields, but later, *Aphis spiraecola* and *Aphis gossypii* have become the most abundant. The change in the aphid dominant populations may be linked with climate change and multiple human factors. Aphids exhibit small sizes with varied life cycles, and few distinct morphological characteristics (Zhang and Zhong 1983; Blackman and Eastop 2006). The classification of *A.* (*A.*) *spiraecola* has always taxonomic inconsistency with *Aphis citricola* (Neubauer et al. 1981; Zhang and Zhong 1983; Lykouressis 1990), due to their shared primary hosts citrus species. Consequently, *Aphis citricola* Van der Goot is an alternative name (Andreev et al. 2009). However, *A.* (*A.*) *spiraecola* can be considered the legitimate valid scientific name (Blackman and Eastop 2006; Yu and Wang 2019). Additionally, *A.* (*A.*) *spiraecola* also exhibits the significant similarity with *Aphis pomi*, and both species have been identified as apple pests. Their morphological similarities result in taxonomic inconsistency; nevertheless, it is imperative to recognize that both are the two distinct species of aphids (Baker and Turner 1916; Blackman and Eastop 2006; Foottit et al. 2009; Rakauskas et al. 2015).

*Aphis* (*Toxoptera*) *citricidus* has consistently been referenced in many studies as *A.* (*T.*) *citricida* (Michaud and Belliure 2000; Amalendu et al. 2015; Zhao et al. 2021). However, this nomenclature may be considered invalid (Nafría et al. 2005; Blackman and Eastop 2006). Nafría et al. (2005) demonstrated the detailed information about the scientific name of *A.* (*T.*) *citricidus*, and finally concluded the name *Toxoptera citricida* constitutes an unjustified emendation by Stoetzel. Furthermore, *A.* (*T.*) *citricidus* was considered to be a subgenus of *Aphis* (Lagos et al. 2014), whereas, in the latest study, it has been proposed that *Toxoptera citricidus* should revert to *Aphis citricidus* based on the phylogenetic analysis (Wei et al. 2019). The present study aims to synthesize these perspectives, advocating for the classification of *Toxoptera* as a subgenus, due to its morphometric similarities to *Aphis*, particularly stridulatory apparatus consisting of ventrolateral ridges on the abdomen and a row of peg-like hairs (Zhang and Zhong 1983; Blackman and Eastop 2006). As prevalent and concerning pests in citriculture, multiple aphid species, including *A.* (*A.*) *gossypii*, *A.* (*A.*) *spiraecola*, *A.* (*T.*) *aurantii*, and *A.* (*T.*) *citricidus*, are associated with citrus tristeza virus (CTV) (Rocha-Peña et al. 1995; Zhang 2000; Brlansky et al. 2011). Furthermore, citrus yellow vein clearing virus (CYVCV) has also been associated with *A.* (*A.*) *gossypii* and *A.* (*A.*) *spiraecola*, which have become prevalent in citrus plantations across China during recent years (Zhang et al. 2017, 2018; Liu 2018; Afloukou et al. 2021). *A. *(*T.*) *citricidus* (Kirkaldy), commonly known as the brown citrus aphid, is widely recognized as one of the most destructive aphid species infesting citrus crops globally, primarily due to its exceptional efficiency as a vector of CTV (Yokomi et al. 1994). Additionally, *Aphis* (*A.*) *gossypii* (Glover), the cotton aphid, has also demonstrated considerable efficiency in transmitting CTV, particularly in citrus-growing regions such as California, Israel, and Spain (Cambra et al. 2000; Marroquín et al. 2004; Yokomi and DeBorde 2005). Conversely, *A*. (*T*.) *aurantii* (Boyer de Fonscolombe) and *A.* (*A.*) *spiraecola* Patch are generally regarded as less competent vectors (Norman and Grant 1956). It is noteworthy that beyond species-specific transmission efficiencies, environmental and ecological factors such as citrus flush cycles, host plant vigor, and barriers to aphid dispersal can significantly influence aphid population dynamics and, consequently, the epidemiology of CTV. These dynamics may vary markedly even among orchards within the same geographical zone. Importantly, prior surveys conducted in Mediterranean regions, such as Greece, have documented the presence of nine aphid species associated with citrus (Kalaitzak et al. 2019), yet notably, none of these included *A*. (*T*.) *citricidus*. Our findings, therefore, not only update the taxonomic records for Zhejiang Province but also underscore the importance of region-specific surveillance in understanding the vector potential and distribution of citrus aphids. Furthermore, the present investigation unveiled the important morphological and molecular aspects of key citrus aphid species These results enrich our knowledge of aphid dynamics and identification of putative viruliferous vectors, facilitating the development of sustainable approaches for the control of both aphids and CTV. And future research should prioritize the investigation of the relationship between aphids and citrus viruses, alongside the development of improved management strategies.

## Supplementary Information

Below is the link to the electronic supplementary material.

ESM 1 (DOCX 54.0 KB)

## Declarations

This study did not involve human or animal subjects, and thus, no ethical approval was required. The study protocol adhered to the guidelines established by the journal.
