# Supplementary material for: Investigating the Citrus Aphid Species in Zhejiang, China: Morphometric Analysis and Genetic Distinctions
Source: Neotrop Entomol. 2025 Jul 10;54(1):83. doi: 10.1007/s13744-025-01299-x (PMC12246005; doi:10.1007/s13744-025-01299-x)
Supplement: Supplementary file 1 — (DOCX 54.0 KB) [file 13744_2025_1299_MOESM1_ESM.docx]

**Supplementary materials**

*Measurements follow standard taxonomic protocols (Blackman & Eastop, 2000). BL: Body length; ANT III–VI: Antennal segments; R IV+V: Rostrum length; HT II: Hind tibia length; Siphunculi/Cauda: Basal width + length. Data represent individual slide specimens (n=11–15 per species).

**Table S1. The morphological parameters of *Aphis* (*Aphis*) *craccivora* (n=12)***

| BL | ANT | | | | R Ⅳ + Ⅴ | HT Ⅱ | Siphunculi  (Basal width + Length) | Cauda  (Basal width + Length) |
| --- | --- | --- | --- | --- | --- | --- | --- | --- |
|  | Ⅲ | Ⅳ | Ⅴ | Ⅵ  (PT + Base) |  |  |  |  |
| 1.60 | 0.20 | 0.20 | 0.15 | 0.21 + 0.11 | 0.10 | 0.09 | 0.09 + 0.28 | 0.09 + 0.16 |
| 1.41 | 0.27 | 0.18 | 0.16 | 0.23 + 0.11 | 0.12 | 0.10 | 0.09 + 0.30 | 0.09 + 0.16 |
| 1.38 | 0.26 | 0.14 | 0.16 | 0.23 + 0.09 | 0.12 | 0.09 | 0.07 + 0.30 | 0.09 + 0.16 |
| 1.35 | 0.23 | 0.17 | 0.14 | 0.23 + 0.10 | 0.12 | 0.10 | 0.09 + 0.29 | 0.09 + 0.16 |
| 1.41 | 0.31 | 0.20 | 0.19 | 0.28 + 0.12 | 0.09 | 0.11 | 0.11 + 0.37 | 0.11 + 0.23 |
| 1.52 | 0.27 | 0.18 | 0.16 | 0.28 + 0.09 | 0.09 | 0.10 | 0.10 + 0.31 | 0.12 + 0.19 |
| 1.58 | 0.27 | 0.20 | 0.19 | 0.30 + 0.11 | 0.10 | 0.10 | 0.10 + 0.35 | 0.12 + 0.19 |
| 1.40 | 0.31 | 0.23 | 0.19 | 0.27 + 0.10 | 0.10 | 0.11 | 0.10 + 0.43 | 0.10 + 0.21 |
| 1.44 | 0.30 | 0.20 | 0.17 | 0.26 + 0.09 | 0.10 | 0.10 | 0.10 + 0.33 | 0.12 + 0.19 |
| 1.51 | 0.30 | 0.21 | 0.18 | 0.30 + 0.10 | 0.11 | 0.11 | 0.11 + 0.36 | 0.11 + 0.21 |
| 1.37 | 0.25 | 0.14 | 0.14 | 0.20 + 0.08 | 0.08 | 0.08 | 0.08 + 0.26 | 0.09 + 0.16 |
| 1.37 | 0.28 | 0.20 | 0.17 | 0.24 + 0.09 | 0.09 | 0.10 | 0.10 + 0.33 | 0.09 + 0.20 |

* each row in the table represents a citrus aphid slide sample.

**Table S2. The morphological parameters of *Aphis* (*Aphis*) *gossypii* (n=15)***

| BL | ANT | | | | R Ⅳ + Ⅴ | HT Ⅱ | Siphunculi  (Basal width + Length) | Cauda  (Basal width + Length) |
| --- | --- | --- | --- | --- | --- | --- | --- | --- |
|  | Ⅲ | Ⅳ | Ⅴ | Ⅵ  (PT + Base) |  |  |  |  |
| 1.37 | 0.23 | 0.17 | 0.15 | 0.29 + 0.09 | 0.10 | 0.08 | 0.10 + 0.28 | 0.08 + 0.15 |
| 1.39 | 0.24 | 0.16 | 0.16 | 0.29 + 0.10 | 0.11 | 0.09 | 0.10 + 0.29 | 0.09 + 0.14 |
| 1.48 | 0.25 | 0.17 | 0.17 | 0.31 + 0.10 | 0.09 | 0.08 | 0.10 + 0.31 | 0.07 + 0.15 |
| 1.40 | 0.28 | 0.19 | 0.17 | 0.30 + 0.11 | 0.11 | 0.09 | 0.11 + 0.37 | 0.12 + 0.17 |
| 1.49 | 0.29 | 0.20 | 0.17 | 0.30 + 0.10 | 0.11 | 0.10 | 0.11 + 0.36 | 0.10 + 0.15 |
| 1.46 | 0.31 | 0.21 | 0.19 | 0.30 + 0.10 | 0.11 | 0.10 | 0.12 + 0.39 | 0.10 + 0.17 |
| 1.48 | 0.27 | 0.18 | 0.17 | 0.29 + 0.09 | 0.10 | 0.10 | 0.11 + 0.36 | 0.10 + 0.17 |
| 1.39 | 0.23 | 0.15 | 0.13 | 0.29 + 0.09 | 0.10 | 0.09 | 0.10 + 0.28 | 0.08 + 0.13 |
| 1.35 | 0.21 | 0.15 | 0.14 | 0.28 + 0.09 | 0.10 | 0.08 | 0.09 + 0.30 | 0.08 + 0.14 |
| 1.43 | 0.23 | 0.15 | 0.13 | 0.29 + 0.09 | 0.10 | 0.10 | 0.09 + 0.32 | 0.08 + 0.14 |
| 1.44 | 0.23 | 0.15 | 0.14 | 0.25 + 0.10 | 0.10 | 0.09 | 0.09 + 0.31 | 0.08 + 0.15 |
| 1.36 | 0.24 | 0.14 | 0.13 | 0.25 + 0.08 | 0.10 | 0.09 | 0.09 + 0.28 | 0.08 + 0.15 |
| 1.34 | 0.26 | 0.17 | 0.15 | 0.27 + 0.11 | 0.11 | 0.08 | 0.10 + 0.32 | 0.08 + 0.14 |
| 1.39 | 0.25 | 0.15 | 0.13 | 0.29 + 0.09 | 0.11 | 0.08 | 0.10 + 0.32 | 0.09 + 0.14 |
| 1.44 | 0.22 | 0.15 | 0.14 | 0.26 + 0.10 | 0.10 | 0.09 | 0.10 + 0.30 | 0.09 + 0.15 |

* each row in the table represents a citrus aphid slide sample.

**Table S3. The morphological parameters of *Aphis* (*Aphis*) *spiraecola* (n=14)***

| BL | ANT | | | | R Ⅳ + Ⅴ | HT Ⅱ | Siphunculi  (Basal width + Length) | Cauda  (Basal width + Length) |
| --- | --- | --- | --- | --- | --- | --- | --- | --- |
|  | Ⅲ | Ⅳ | Ⅴ | Ⅵ  (PT + Base) |  |  |  |  |
| 1.54 | 0.26 | 0.19 | 0.16 | 0.26 + 0.09 | 0.11 | 0.09 | 0.09 + 0.34 | 0.13 + 0.20 |
| 1.43 | 0.27 | 0.17 | 0.16 | 0.21 + 0.08 | 0.11 | 0.09 | 0.09 + 0.29 | 0.09 + 0.18 |
| 1.59 | 0.25 | 0.19 | 0.17 | 0.24 + 0.09 | 0.11 | 0.09 | 0.10 + 0.34 | 0.11 + 0.18 |
| 1.51 | 0.26 | 0.19 | 0.16 | 0.26 + 0.09 | 0.11 | 0.09 | 0.09 + 0.37 | 0.12 + 0.20 |
| 1.46 | 0.28 | 0.20 | 0.17 | 0.26 + 0.09 | 0.10 | 0.09 | 0.10 + 0.36 | 0.09 + 0.18 |
| 1.62 | 0.24 | 0.18 | 0.14 | 0.24 + 0.10 | 0.11 | 0.09 | 0.08 + 0.30 | 0.09 + 0.19 |
| 1.45 | 0.27 | 0.21 | 0.16 | 0.27 + 0.10 | 0.11 | 0.09 | 0.09 + 0.34 | 0.10 + 0.19 |
| 1.60 | 0.27 | 0.22 | 0.16 | 0.27 + 0.11 | 0.11 | 0.10 | 0.08 + 0.34 | 0.12 + 0.20 |
| 1.52 | 0.25 | 0.18 | 0.16 | 0.22 + 0.10 | 0.10 | 0.09 | 0.10 + 0.34 | 0.11 + 0.19 |
| 1.57 | 0.27 | 0.18 | 0.17 | 0.26 + 0.10 | 0.12 | 0.10 | 0.11 + 0.34 | 0.13 + 0.19 |
| 1.42 | 0.27 | 0.20 | 0.16 | 0.25 + 0.11 | 0.11 | 0.10 | 0.09 + 0.31 | 0.11 + 0.18 |
| 1.41 | 0.26 | 0.17 | 0.16 | 0.25 + 0.09 | 0.12 | 0.10 | 0.09 + 0.33 | 0.13 + 0.18 |
| 1.42 | 0.25 | 0.19 | 0.16 | 0.24 + 0.10 | 0.10 | 0.10 | 0.09 + 0.31 | 0.13 + 0.17 |
| 1.50 | 0.21 | 0.16 | 0.13 | 0.21 + 0.10 | 0.10 | 0.09 | 0.10 + 0.25 | 0.10 + 0.17 |

* each row in the table represents a citrus aphid slide sample.

**Table S4. The morphological parameters of *Aphis* (*Toxoptera*) *aurantii* (n=11)***

| BL | ANT | | | | R Ⅳ + Ⅴ | HT Ⅱ | Siphunculi  (Basal width + Length) | Cauda  (Basal width + Length) |
| --- | --- | --- | --- | --- | --- | --- | --- | --- |
|  | Ⅲ | Ⅳ | Ⅴ | Ⅵ  (PT + Base) |  |  |  |  |
| 1.66 | 0.32 | 0.29 | 0.30 | 0.42 + 0.10 | 0.12 | 0.09 | 0.14 + 0.29 | 0.11 + 0.22 |
| 1.63 | 0.30 | 0.23 | 0.23 | 0.34 + 0.08 | 0.12 | 0.08 | 0.11 + 0.23 | 0.11 + 0.21 |
| 1.68 | 0.30 | 0.23 | 0.23 | 0.41 + 0.09 | 0.12 | 0.07 | 0.13 + 0.24 | 0.11 + 0.21 |
| 1.51 | 0.34 | 0.26 | 0.26 | 0.41 + 0.10 | 0.12 | 0.08 | 0.12 + 0.26 | 0.12 + 0.22 |
| 1.48 | 0.33 | 0.25 | 0.25 | 0.40 + 0.09 | 0.12 | 0.09 | 0.12 + 0.25 | 0.10 + 0.22 |
| 1.42 | 0.33 | 0.26 | 0.26 | 0.42 + 0.10 | 0.13 | 0.09 | 0.13 + 0.27 | 0.10 + 0.21 |
| 1.51 | 0.28 | 0.21 | 0.22 | 0.36 + 0.08 | 0.12 | 0.08 | 0.12 + 0.23 | 0.11 + 0.21 |
| 1.58 | 0.35 | 0.29 | 0.28 | 0.43 + 0.10 | 0.12 | 0.08 | 0.12 + 0.27 | 0.10 + 0.23 |
| 1.69 | 0.26 | 0.24 | 0.27 | 0.43 + 0.10 | 0.13 | 0.07 | 0.11 + 0.23 | 0.11 + 0.19 |
| 1.69 | 0.31 | 0.27 | 0.28 | 0.44 + 0.09 | 0.13 | 0.08 | 0.11 + 0.26 | 0.10 + 0.17 |
| 1.63 | 0.30 | 0.24 | 0.24 | 0.39 + 0.08 | 0.13 | 0.07 | 0.10 + 0.24 | 0.09 + 0.20 |

* each row in the table represents a citrus aphid slide sample.

**Table S5. The morphological parameters of *Aphis* (*Toxoptera*) *citricidus* (n=12)***

| BL | ANT | | | | R Ⅳ + Ⅴ | HT Ⅱ | Siphunculi  (Basal width + Length) | Cauda  (Basal width + Length) |
| --- | --- | --- | --- | --- | --- | --- | --- | --- |
|  | Ⅲ | Ⅳ | Ⅴ | Ⅵ  (PT + Base) |  |  |  |  |
| 2.16 | 0.46 | 0.37 | 0.27 | 0.54 + 0.10 | 0.15 | 0.10 | 0.15 + 0.39 | 0.16 + 0.28 |
| 2.00 | 0.43 | 0.33 | 0.24 | 0.44 + 0.09 | 0.13 | 0.10 | 0.15 + 0.38 | 0.17 + 0.26 |
| 2.04 | 0.41 | 0.34 | 0.28 | 0.54 + 0.09 | 0.15 | 0.10 | 0.14 + 0.39 | 0.16 + 0.30 |
| 2.15 | 0.42 | 0.38 | 0.28 | 0.55 + 0.11 | 0.15 | 0.10 | 0.14 + 0.43 | 0.17 + 0.28 |
| 2.13 | 0.41 | 0.36 | 0.24 | 0.50 + 0.11 | 0.15 | 0.10 | 0.14 + 0.37 | 0.18 + 0.27 |
| 2.24 | 0.50 | 0.42 | 0.30 | 0.56 + 0.11 | 0.16 | 0.10 | 0.13 + 0.42 | 0.17 + 0.29 |
| 2.24 | 0.42 | 0.35 | 0.26 | 0.56 + 0.10 | 0.15 | 0.11 | 0.16 + 0.40 | 0.20 + 0.28 |
| 2.17 | 0.43 | 0.34 | 0.21 | 0.47 + 0.10 | 0.14 | 0.12 | 0.15 + 0.39 | 0.19 + 0.30 |
| 2.02 | 0.35 | 0.28 | 0.21 | 0.45 + 0.10 | 0.14 | 0.11 | 0.14 + 0.32 | 0.14 + 0.28 |
| 1.96 | 0.39 | 0.27 | 0.22 | 0.44 + 0.08 | 0.14 | 0.11 | 0.16 + 0.33 | 0.17 + 0.28 |
| 2.09 | 0.38 | 0.25 | 0.21 | 0.41 + 0.08 | 0.13 | 0.10 | 0.15 + 0.31 | 0.14 + 0.27 |
| 2.10 | 0.37 | 0.29 | 0.22 | 0.47 + 0.09 | 0.14 | 0.11 | 0.16 + 0.33 | 0.13 + 0.29 |

* each row in the table represents a citrus aphid slide sample.

**Table S6.** **Voucher information of the citrus aphids examined from Zhejiang Province in this study**

| Species | Voucher number | Host plant | Information |
| --- | --- | --- | --- |
| *Aphis* (*Aphis*) *craccivora* | L17 | *Citrus* | Taizhou City, Huangyan District, Toutuo Town  28°64′62″N, 121°16′94″E |
|  | L66 | *Citrus* | Taizhou City, Tiantai County, Baihe Town  29°19′00″N, 120°95′49″E |
| *Aphis* (*Aphis*) *gossypii* | L430 | *Citrus* | Lishui City, Jinyun County, Dongdu Town  28°61′93″N, 120°10′37″E |
|  | L436 | *Citrus* | Lishui City, Jinyun County, Xinjian Town  28°73′00″N, 120°06′12″E |
|  | L324 | *Citrus* | Ningbo City, Xiangshan County, Xiaotang Town  29°21′12″N, 121°84′35″E |
|  | L287 | *Citrus* | Quzhou City, Kecheng District, Shiliang Town  29°00′83″N, 118°80′60″E |
|  | L8 | *Citrus* | Taizhou City, Huangyan District, Toutuo Town  28°64′62″N, 121°16′94″E |
|  | L68 | *Citrus* | Taizhou City, Tiantai County, Baihe Town  29°19′00″N, 120°95′49″E |
| *Aphis* (*Aphis*) *spiraecola* | L21 | *Citrus* | Lishui City, Liandou District, Taiping Town  28°54′47″N, 119°87′95″E |
|  | L274 | *Citrus* | Lishui City, Qingyuan County, Zhukou Town  27°67′24″N, 118°92′48″E |
|  | L322 | *Citrus* | Ningbo City, Xiangshan County, Xiaotang Town  29°21′12″N, 121°84′35″E |
|  | L133 | *Citrus* | Quzhou City, Qujiang District, Lianhua Town  29°09′79″N, 119°01′01″E |
|  | L144 | *Citrus* | Quzhou City, Kecheng District, Shiliang Town  29°00′50″N, 118°81′44″E |
|  | L15 | *Citrus* | Taizhou City, Huangyan District, Yongning Park  28°66′07″N, 121°26′02″E |
|  | L120 | *Citrus* | Taizhou City, Wenling City, Wugen Town  28°33′55″N, 121°25′37″E |
|  | L390 | *Citrus* | Wenzhou City, Pingyang County, Kunyang Town  27°68′31″N, 120°53′64″E |
| *Aphis* (*Toxoptera*) *aurantii* | L11 | *Citrus* | Taizhou City, Huangyan District, Toutuo Town  28°64′62″N, 121°16′94″E |
|  | L246 | *Citrus* | Taizhou City, Huangyan District, Toutuo Town  28°64′58″N, 121°16′88″E |
| *Aphis* (*Toxoptera*) *citricidus* | L142 | *Citrus* | Quzhou City, Kecheng District, Huashu Town  28°86′44″N, 118°75′90″E |
|  | L151 | *Citrus* | Quzhou City, Changshan County, Tonggong Town  28°91′14″N, 118°43′48″E |
|  | L43 | *Citrus* | Taizhou City, Huangyan District, Toutuo Town  28°63′99″N, 121°17′32″E |

**Table S7. Sequences of the aphid species examined in the phylogenetic analysis**

| Species | GenBank Accession No. |
| --- | --- |
| *Aphis* (*Aphis*) *spiraecola* | MT445566, NC_053819, AB506735 |
| *Aphis* (*Aphis*) *craccivora* | KY322895, MN320211 |
| *Aphis* (*Toxoptera*) *aurantii* | KR856183, MN397939, NC_052865, MT850295 |
| *Aphis* (*Toxoptera*) *citricidus* | OM552038, NC_043903, MT296818, EU701936 |
| *Aphis* (*Aphis*) *gossypii* | PP349796, KR017753, KY679347, MH215200, MH407718, MN320089, MT430940 |
| *Myzus persicae* | KY509874 |
| *Diuraphis noxia* | AF548467 |

**Table S8. Genetic distances within and between the *Aphis* (*Aphis*) *craccivora* clade based on the COI gene**

| No.* | 1 | 2 | 3 | 4 | 5 | 6 |
| --- | --- | --- | --- | --- | --- | --- |
| 1 |  |  |  |  |  |  |
| 2 | 0.000 |  |  |  |  |  |
| 3 | 0.000 | 0.000 |  |  |  |  |
| 4 | 0.000 | 0.000 | 0.000 |  |  |  |
| 5 | 0.819 | 0.819 | 0.819 | 0.819 |  |  |
| 6 | 0.821 | 0.821 | 0.821 | 0.821 | 0.084 |  |

*Notes: 1 L17; 2 L66; 3 *Aphis craccivora* KY322895; 4 *Aphis craccivora* MN320211; 5 *Myzus persicae* KY509874; 6 *Diuraphis noxia* AF548467.

**Table S9. Genetic distances within and between the *Aphis* (*Aphis*) *gossypii* clade based on the COI gene**

| No. * | 1 | 2 | 3 | 4 | 5 | 6 | 7 | 8 | 9 | 10 | 11 | 12 | 13 | 14 | 15 |
| --- | --- | --- | --- | --- | --- | --- | --- | --- | --- | --- | --- | --- | --- | --- | --- |
| 1 |  |  |  |  |  |  |  |  |  |  |  |  |  |  |  |
| 2 | 0.000 |  |  |  |  |  |  |  |  |  |  |  |  |  |  |
| 3 | 0.000 | 0.000 |  |  |  |  |  |  |  |  |  |  |  |  |  |
| 4 | 0.000 | 0.000 | 0.000 |  |  |  |  |  |  |  |  |  |  |  |  |
| 5 | 0.000 | 0.000 | 0.000 | 0.000 |  |  |  |  |  |  |  |  |  |  |  |
| 6 | 0.000 | 0.000 | 0.000 | 0.000 | 0.000 |  |  |  |  |  |  |  |  |  |  |
| 7 | 0.000 | 0.000 | 0.000 | 0.000 | 0.000 | 0.000 |  |  |  |  |  |  |  |  |  |
| 8 | 0.000 | 0.000 | 0.000 | 0.000 | 0.000 | 0.000 | 0.000 |  |  |  |  |  |  |  |  |
| 9 | 0.000 | 0.000 | 0.000 | 0.000 | 0.000 | 0.000 | 0.000 | 0.000 |  |  |  |  |  |  |  |
| 10 | 0.000 | 0.000 | 0.000 | 0.000 | 0.000 | 0.000 | 0.000 | 0.000 | 0.000 |  |  |  |  |  |  |
| 11 | 0.000 | 0.000 | 0.000 | 0.000 | 0.000 | 0.000 | 0.000 | 0.000 | 0.000 | 0.000 |  |  |  |  |  |
| 12 | 0.000 | 0.000 | 0.000 | 0.000 | 0.000 | 0.000 | 0.000 | 0.000 | 0.000 | 0.000 | 0.000 |  |  |  |  |
| 13 | 0.000 | 0.000 | 0.000 | 0.000 | 0.000 | 0.000 | 0.000 | 0.000 | 0.000 | 0.000 | 0.000 | 0.000 |  |  |  |
| 14 | 0.809 | 0.809 | 0.809 | 0.809 | 0.809 | 0.809 | 0.809 | 0.809 | 0.809 | 0.809 | 0.809 | 0.809 | 0.809 |  |  |
| 15 | 0.783 | 0.783 | 0.783 | 0.783 | 0.783 | 0.783 | 0.783 | 0.783 | 0.783 | 0.783 | 0.783 | 0.783 | 0.783 | 0.084 |  |

*Notes: 1 L8; 2 L68; 3 L287; 4 L324; 5 L430; 6 L436; 7 *Aphis gossypii* KR017753; 8 *Aphis gossypii* KY679347; 9 *Aphis gossypii* MH215200; 10 *Aphis gossypii* MH407718; 11 *Aphis gossypii* MN320089; 12 *Aphis gossypii* MT430940; 13 *Aphis gossypii* PP349796; 14 *Myzus persicae* KY509874; 15 *Diuraphis noxia* AF548467.

**Table S10. Genetic distances within and between the *Aphis* (*Aphis*) *spiraecola* clade based on the COI gene**

| No. * | 1 | 2 | 3 | 4 | 5 | 6 | 7 | 8 | 9 | 10 | 11 | 12 | 13 |
| --- | --- | --- | --- | --- | --- | --- | --- | --- | --- | --- | --- | --- | --- |
| 1 |  |  |  |  |  |  |  |  |  |  |  |  |  |
| 2 | 0.002 |  |  |  |  |  |  |  |  |  |  |  |  |
| 3 | 0.000 | 0.002 |  |  |  |  |  |  |  |  |  |  |  |
| 4 | 0.000 | 0.002 | 0.000 |  |  |  |  |  |  |  |  |  |  |
| 5 | 0.000 | 0.002 | 0.000 | 0.000 |  |  |  |  |  |  |  |  |  |
| 6 | 0.000 | 0.002 | 0.000 | 0.000 | 0.000 |  |  |  |  |  |  |  |  |
| 7 | 0.000 | 0.002 | 0.000 | 0.000 | 0.000 | 0.000 |  |  |  |  |  |  |  |
| 8 | 0.000 | 0.002 | 0.000 | 0.000 | 0.000 | 0.000 | 0.000 |  |  |  |  |  |  |
| 9 | 0.000 | 0.002 | 0.000 | 0.000 | 0.000 | 0.000 | 0.000 | 0.000 |  |  |  |  |  |
| 10 | 0.000 | 0.002 | 0.000 | 0.000 | 0.000 | 0.000 | 0.000 | 0.000 | 0.000 |  |  |  |  |
| 11 | 0.000 | 0.002 | 0.000 | 0.000 | 0.000 | 0.000 | 0.000 | 0.000 | 0.000 | 0.000 |  |  |  |
| 12 | 0.833 | 0.829 | 0.833 | 0.833 | 0.833 | 0.833 | 0.833 | 0.833 | 0.833 | 0.833 | 0.833 |  |  |
| 13 | 0.818 | 0.813 | 0.818 | 0.818 | 0.818 | 0.818 | 0.818 | 0.818 | 0.818 | 0.818 | 0.818 | 0.084 |  |

*Notes: 1 L15; 2 L21; 3 L120; 4 L133; 5 L144; 6 L274; 7 L322; 8 L390; 9 *Aphis spiraecola* AB506735; 10 *Aphis spiraecola* MT445566; 11 *Aphis spiraecola* NC_053819; 12 *Myzus persicae* KY509874; 13 *Diuraphis noxia* AF548467.

**Table S11. Genetic distances within and between the *Aphis* (*Toxoptera*) *aurantii* clade based on the COI gene**

| No. * | 1 | 2 | 3 | 4 | 5 | 6 | 7 | 8 |
| --- | --- | --- | --- | --- | --- | --- | --- | --- |
| 1 |  |  |  |  |  |  |  |  |
| 2 | 0.000 |  |  |  |  |  |  |  |
| 3 | 0.000 | 0.000 |  |  |  |  |  |  |
| 4 | 0.003 | 0.003 | 0.003 |  |  |  |  |  |
| 5 | 0.003 | 0.003 | 0.003 | 0.003 |  |  |  |  |
| 6 | 0.002 | 0.002 | 0.002 | 0.002 | 0.002 |  |  |  |
| 7 | 0.816 | 0.816 | 0.816 | 0.805 | 0.805 | 0.810 |  |  |
| 8 | 0.816 | 0.816 | 0.816 | 0.810 | 0.805 | 0.810 | 0.084 |  |

*Notes: 1 L11; 2 L246; 3 *Aphis aurantii* KR856183; 4 *Aphis aurantii* MN397939; 5 *Aphis aurantii* MT850295; 6 *Aphis aurantii* NC_052865; 7 *Myzus persicae* KY509874; 8 *Diuraphis noxia* AF548467.

**Table S12. Genetic distances within and between the *Aphis* (*Toxoptera*) *citricidus* clade based on the COI gene**

| No. * | 1 | 2 | 3 | 4 | 5 | 6 | 7 | 8 | 9 |
| --- | --- | --- | --- | --- | --- | --- | --- | --- | --- |
| 1 |  |  |  |  |  |  |  |  |  |
| 2 | 0.002 |  |  |  |  |  |  |  |  |
| 3 | 0.002 | 0.000 |  |  |  |  |  |  |  |
| 4 | 0.002 | 0.000 | 0.000 |  |  |  |  |  |  |
| 5 | 0.002 | 0.000 | 0.000 | 0.000 |  |  |  |  |  |
| 6 | 0.005 | 0.003 | 0.003 | 0.003 | 0.003 |  |  |  |  |
| 7 | 0.031 | 0.029 | 0.029 | 0.029 | 0.029 | 0.033 |  |  |  |
| 8 | 0.845 | 0.840 | 0.840 | 0.840 | 0.840 | 0.845 | 0.813 |  |  |
| 9 | 0.845 | 0.840 | 0.840 | 0.840 | 0.840 | 0.845 | 0.803 | 0.084 |  |

*Notes: 1 L43; 2 L142; 3 L151; 4 *Toxoptera citricida* EU701936; 5 *Aphis citricidus* MT296818; 6 *Aphis citricidus* NC_043903; 7 *Aphis citricidus* OM552038; 8 *Myzus persicae* KY509874; 9 *Diuraphis noxia* AF548467.
